# The Impact of Breast Reduction Practice Variation in Hamilton, Ontario: A Cost Analysis

**DOI:** 10.1177/22925503251386770

**Published:** 2025-10-21

**Authors:** Tara Behroozian, Alice Wang, Lucas Gallo, Justin Haas, Helene Retrouvey, Ammara Ghumman, Achilles Thoma

**Affiliations:** 1 Division of Plastic Surgery, Department of Surgery, 3710 McMaster University, Hamilton, Ontario, Canada; 2 Department of Medicine, 3710 McMaster University, Hamilton, Ontario, Canada; 3 Department of Health Research Methods, Evidence and Impact (HEI), 3710 McMaster University, Hamilton, Ontario, Canada

**Keywords:** bilateral breast reduction, reduction mammaplasty, breast hypertrophy, cost analysis, practice variations, réduction mammaire bilatérale, mammoplastie de réduction, hypertrophie mammaire,, analyse de coût / évaluation économique, variation de pratiques

## Abstract

**Introduction:** Bilateral breast reduction (BBR) is one of the most common Ministry of Health-funded plastic surgery procedures. Surgeon variation in BBR management has been reported. In this study, we aimed to answer the following: what are the current variations in plastic surgeons’ management of BBR in Hamilton, Ontario?, and what are the cost implications of these practice variations to the healthcare system and patients? **Methods:** An online survey was conducted to gather demographic and practice data from all plastic surgeons practicing in Hamilton from 2019 to 2024. Reference costs for pre-, intra-, and postoperative components of BBR were obtained from a tertiary academic care center, the Ontario Schedule of Benefits for Physician Services, and local nursing care agencies, and used in the calculation of individual surgeons’ healthcare and patient costs. **Results:** Among 17 plastic surgeons practicing in Hamilton, 9 perform BBR, and 7 participated in the survey (response rate = 77.8%). Variations in practice were identified across several areas, including drain use, nursing care, OR time, and frequency of physician appointments. The mean healthcare costs per BBR patient were $5156 and $7146 for the lowest- and highest-costing surgeons, respectively, resulting in a difference of up to $2034 per patient. Patient-borne costs varied from $92 to $179 between surgical practices. **Conclusion:** There is considerable variability in BBR practice among plastic surgeons in Hamilton, contributing to a difference in overall healthcare and patient costs. Given the importance of cost savings in a single-payer system, future research is warranted to standardize BBR practices.

## Résumé

**Introduction :** La réduction mammaire bilatérale (RMB) est l’une des procédures de chirurgie plastique les plus courantes financées par le ministère de la Santé. Une variation dans la gestion de la RMB par les chirurgiens a été signalée. Dans cette étude, nous avons cherché à répondre aux questions suivantes : quelles sont les variations actuelles dans la gestion de la RMB par les chirurgiens plasticiens à Hamilton (Ontario) et quelles sont les implications sur les coûts de ces différences entre pratiques pour le système de santé et pour les patients? **Méthodes :** Une enquête en ligne a été effectuée pour rassembler des données démographiques et sur les pratiques de tous les chirurgiens plasticiens exerçant à Hamilton de 2019 à 2024. Les coûts de référence pour les composantes pré-, per- et postopératoires de la RMB ont été obtenues auprès d’un centre universitaire de soins tertiaires, du Barème des prestations et des honoraires de l’Assurance-santé et des agences locales de soins infirmiers; ils ont été utilisés dans le calcul des soins de santé des chirurgiens individuels et des coûts pour les patients. **Résultats :** Parmi les 17 chirurgiens plasticiens exerçant à Hamilton, neuf réalisaient des RMB et sept ont participé à l’enquête (taux de réponse = 77,8%). Les variations pratiques ont été identifiées dans plusieurs domaines, notamment l’utilisation d’un drain, les soins infirmiers, le temps de bloc opératoire et la fréquence des rendez-vous avec le médecin. Les coûts moyens des soins par patiente de RMB étaient de 5 156 $ et de 7 146 $ pour, respectivement, les chirurgiens les moins et les plus coûteux, soit une différence pouvant aller jusqu’à 2 034 $ par patient. Les coûts supportés par la patiente variaient de 92 $ à 179 $ selon les pratiques chirurgicales. **Conclusion :** Il y a une variabilité considérable dans la pratique de la RMB parmi les chirurgiens plasticiens de Hamilton, contribuant à des différences dans les coûts globaux pour le système de santé et pour les patients. Compte tenu de l’importance des économies dans un système à payeur unique, des recherches supplémentaires sont justifiées pour standardiser la pratique de la RMB.

## Introduction

Bilateral breast reduction (BBR) is one of the most commonly performed Ministry of Health (MOH)-funded plastic surgery procedures in Canada, with an estimated 5000 performed per year in Ontario.^1^ Patients are increasingly opting for BBR—with a 54% increase from 2019 to 2022—to address the distressing physical and psychosocial implications of breast hypertrophy.^2^ In Ontario, BBR is indicated and eligible for coverage by the MOH through the Ontario Health Insurance Plan (OHIP) in breast hypertrophy patients who experience related symptoms of pain (back, neck, and/or shoulder), bra-strap grooving, and inframammary skin irritation or intertrigo.^1^

Despite the popularity of BBR, practice patterns vary significantly, with a 2008 survey highlighting variability in Canadian plastic surgeons’ approaches to care.^3^ Variations in surgical practice can be attributed to uncertainty surrounding optimal evidence-based approaches to care.^4^ When we fail to use the optimal approach to manage a surgical problem, and instead opt for a variety of nonevidence-based approaches in surgical practice, we face the risk of increasing healthcare resource waste.^5^ In Canada's single-payer, publicly funded health care system, eliminating healthcare resource waste secondary to practice variation—previously reported to be as high as 30%—should be our highest priority.^6^

It is recommended that plastic surgeons practicing in our current economic environment prioritize cost savings and consider the use of economic evaluations to guide decision-making when multiple surgical techniques are equally effective.^5^ Choosing a less cost-effective option comes with a risk of redistributions of funding, especially as we face worsening shortages in available healthcare spending dollars.^5^ With such a finite amount of health care resources, plastic surgeons must therefore demonstrate to governing agencies and taxpayers that our practice is rooted in evidence-based guidelines that simultaneously seek to lower healthcare costs while improving patient outcomes and satisfaction.^5^

To date, a BBR-focused economic evaluation within a Canadian setting has not been conducted. Given the significant health burden of breast hypertrophy, the rising demand for BBR surgery, and the variability in BBR clinical practice, it is imperative that approaches to BBR be standardized and cost-effective. In this study, we aim to answer the following questions: (1) What are current variations in approaches to BBR among plastic surgeons in Hamilton, Ontario?; (2) What are the implications of these practice variations on healthcare costs?; and (3) What are the implications of these practice variations on patient-borne costs?.

## Methods

### Survey on Clinical Practices

A cross-sectional study was conducted to answer the research question. A 30-question online survey was developed in Microsoft Forms (Microsoft 365, 2024) to gather information on clinical practice variability in BBR. Input from a group consisting of 3 plastic surgeons, 2 plastic surgery residents, and one medical student was gathered to pretest the questions for clarity and comprehensiveness. In May 2024, plastic surgeons practicing in Hamilton, Ontario, between 2019 and 2024 were identified and invited to participate. Convenience sampling was used, focusing on surgeons practicing in Hamilton to minimize heterogeneity related to institution-specific protocols. The web-based survey questions were derived through gray literature searching of similar surveys previously conducted^3^ and finalized through group input and consensus. Survey questions included assessing characteristics of plastic surgeons’ clinical practice with regard to preoperative, intraoperative, and postoperative management of BBR patients (Appendix A). As well, surgeons’ demographic information was collected, including gender, years in practice, area of fellowship training (if applicable), and estimated average BBR annual caseload.

### Cost Analysis

Healthcare and patient-borne costs obtained include those associated with the OR, physician billing, community nursing care appointments, antibiotic prescriptions, as well as patient transportation and parking costs to attend appointments (Appendix B). Hourly operating room costs for BBR procedures were obtained from the finance department of the tertiary academic care center, through access to the 2023 to 2024 Ontario Case Costing Initiative dataset. Operative cost was estimated by the finance department to be $1788.63 per hour for all BBR cases, which includes direct costs for OR staffing, supplies, intraoperative medications, operative equipment, and building infrastructure. Physician remuneration costs were obtained from the Ontario MOH's *Schedule of Benefits: Physician Services Under the Health Insurance Act*.^7^ Other healthcare costs were obtained through gray literature searching and by contacting local agencies, such as costs of antibiotic prescriptions, and home care clinic visits for postoperative drain and dressing management. Direct costs absorbed by patients were estimated based on the cost of attending clinic appointments (including transportation costs and parking costs). The average patient costs for attending in-hospital clinic appointments and community wound care appointments were estimated to be $108 to $140 and $57 to $69, respectively.

Based on the costs obtained from the aforementioned sources, a minimum and maximum cost per BBR case was calculated for each surgeon. Then, a mean cost per BBR case was estimated for each surgeon by assuming that 50% of their cases incurred the minimum estimated cost and the remaining 50% incurred the maximum estimated cost. Based on this assumption, the mean cost was calculated as the average of the minimum and maximum costs. The full range is also reported to identify intrasurgeon variability in practices. The intersurgeon range between the minimum and maximum estimated costs per BBR case was used to assess the variability in spending across surgeons.

## Results

### Survey Participation

Among 14 plastic surgeons in Hamilton invited to participate in this study, 9 perform BBR routinely, 7 of which completed the survey (response rate: 77.8%). The participating surgeons have been in practice for an average of 13.6 years (range: 1-43), performing between 5 and over 90 BBR cases annually (mean: 33.1 cases). Five surgeons (71.4%) completed a fellowship in the areas of breast and/or microsurgery. Surgeon characteristics are summarized in Table 1. Five of the 7 surgeons (71.4%) only perform OHIP-funded BBRs, with 2 performing a combination of OHIP-funded and private, liposuction-assisted BBRs (28.6%). There is an almost equal division between surgeons who feel they were adequately remunerated for the procedure through the current OHIP fee schedule (n = 3) and those who disagree (n = 4).

**Table 1.** Survey Participant Demographics.

	n (%)
Gender	
Male	3 (42.9)
Female	4 (57.1)
Length of experience (years)	
0-5	3 (42.9)
6-10	1 (14.3)
11-30	2 (28.6)
31+	1 (14.3)
Fellowship training	
Microsurgery	4 (57.1)
Hand and/or peripheral nerve	5 (71.4)
Breast	1 (14.3)
Head and neck	1 (14.3)
Cosmetic	1 (14.3)
Average number of BBRs performed annually	
10 or fewer	2 (28.6)
11-30	2 (28.6)
31-50	2 (28.6)
51+	1 (14.3)

Abbreviation: BBR, bilateral breast reduction.

### Practice Variability

#### Preoperative Care

Variations in practices across all phases of care are summarized in Table 2. Preoperatively, all surgeons recommend that patients stop smoking a minimum of one month prior to surgery, and 6 surgeons (85.7%) recommend that patients lose weight preoperatively to meet a target BMI of 30 (4 surgeons), 32 to 33 (one surgeon), and 35 (2 surgeons). The surgeons report assessing a patient on 1 to 3 occasions (including the initial consultation visit), with some variation depending on patient factors. Four surgeons routinely request a preoperative anesthesia assessment to be completed for all patients undergoing BBR, while the remaining surgeons reserve these assessments for some patients, depending on their comorbidities. Only one surgeon reports conducting a general physical examination, beyond the breast examination, prior to surgery.

**Table 2.** Practice Variability Among Surgeons.

Preoperative practices	n (%)
Do you perform OHIP-funded or private BBRs?	
OHIP only	5 (71.4)
Both	2 (28.6)
Do you operate on smokers?	
Yes	2 (28.6)
No	5 (71.4)
Do you instruct patients to stop smoking before BBR? If so, at what time point before surgery?	
Yes	7 (100)
1 month preoperatively	4 (57.1)
2 months preoperatively	1 (14.3)
3 months preoperatively	2 (28.6)
No	0 (0)
Do you recommend patients to lose weight preoperatively?	
Yes	6 (85.7)
Target BMI of less than 30	4 (57.1)
Target BMI of less than 32-33	1 (14.3)
Target BMI of less than 35	2 (28.6)
No	1 (14.3)
If weight loss is recommended, do you reassess patients after weight loss for stabilization prior to surgery?	
Yes	4 (57.1)
No	2 (28.6)
How many times is the patient seen before the day of operation by the surgeon?	
Once	1 (14.3)
Once or twice (depending on patient factors)	3 (42.9)
Twice	2 (28.6)
Thrice	1 (14.3)
Is a preoperative assessment completed by the anesthesia team?	
Yes	4 (57.1)
Sometimes	3 (42.9)
Do you conduct a general physical examination (eg, cardiac, respiratory, and abdominal) in addition to the breast examination prior to surgery?	
Yes	1 (14.3)
Sometimes	2 (28.6)
No	4 (57.1)
Do patients undergo preoperative mammography?	
Yes, in the following circumstances	5 (71.4)
Age ≥40	5 (71.4)
Family history of breast cancer	4 (57.1)
Most recent mammogram over 2 years ago	4 (57.1)
Palpable lump on physical examination	4 (57.1)
Any clinical issues reported	1 (14.3)
No	2 (28.6)
Do you offer liposuction to patients?	
Yes, all	1 (14.3)
Yes, some	3 (42.9)
No	3 (42.9)

Intraoperative practices	n (%)
Do you use drains?	
Yes	3 (42.9)
Sometimes	1 (14.3)
No	3 (42.9)
Do you use tumescent fluid in the breast?	
Yes	2 (28.6)
Sometimes	1 (14.3)
No	4 (57.1)
Do you use antibiotics intraoperatively/preoperatively?	
Yes	7 (100)
Ancef	6 (85.7)
Keflex	2 (28.6)
No	0 (0)
Do you use venous thromboembolism prophylaxis?	
Yes	5 (71.4)
TED stockings	2 (28.6)
Other compression devices	2 (28.6)
Subcutaneous heparin	4 (57.1)
No	2 (28.6)
How long do you normally book BBR in the OR schedule?	
2 h	1 (14.3)
2.5 h	2 (28.6)
3 h	4 (57.1)
What percentage of your BBRs is liposuction-assisted?	
None (0%)	3 (42.9)
Few (<5%)	1 (14.3)
Half (50%)	2 (28.6)
All (100%)	1 (14.3)
What percentage of your BBRs is liposuction-only?	
None (0%)	6 (85.7)
Few (<5%)	0 (0)
Half (50%)	1 (14.3)
All (100%)	0 (0)
If your BBRs are liposuction-assisted, is the liposuction used for:	
BBR itself	0 (0)
Lateral chest wall	5 (57.1)
Both	1 (14.3)
Postoperative practices	n (%)
What percentage of your BBRs is done as day surgery procedures?	
90%-94%	1 (14.3)
95%-99%	1 (14.3)
100%	5 (71.4)
What percentage of your BBRs is done as same-day admission procedures?	
0%	6 (85.7)
1%-10%	1 (14.3)
How many postoperative visits is the patient routinely scheduled for?	
1	0 (0)
2	4 (57.1)
3	2 (28.6)
4	0 (0)
5	1 (14.3)
Are patients given home care services routinely?	
Yes	2 (28.6)
Sometimes	1 (14.3)
No	4 (57.1)
If you use drains routinely, who performs drain removal?	
Yes or sometimes	4 (57.1)
Myself/in clinic	2 (28.6)
Community nursing care clinic	3 (42.9)
No drains	3 (42.9)
Do you use antibiotics postoperatively?	
Yes	4 (57.1)
Keflex	4 (57.1)
No	3 (42.9)
Are patients given a prescription for compression stockings?	
Yes	1 (14.3)
No	6 (85.7)
Are patients given a prescription for postoperative anticoagulation at home?	
Yes	0 (0)
No	7 (100)

Abbreviations: BBR, bilateral breast reduction; OHIP, Ontario Health Insurance Plan.

#### Intraoperative Care

On average, the mean individual case duration was 2.7 h (range: 2-3) based on the time booked for the OR. Intraoperatively, an equal number of surgeons use drains (42.9%) compared to those who do not (42.9%), while one surgeon reports variable use. Tumescent fluid is always administered by 2 surgeons (28.6%) and occasionally by another surgeon. Antibiotics are given preoperatively or intraoperatively by all surgeons, with cefazolin being the most popular choice, followed by cefalexin. Five surgeons (71.4%) take prophylactic precautions against venous thromboembolism, with subcutaneous heparin being the top modality. Four surgeons (57.1%) offer liposuction to their patients, of which there is significant variation noted in the proportion of patients who actually receive liposuction-assisted BBR. One surgeon exclusively performs liposuction-assisted BBR in all patients.

#### Postoperative Care

Bilateral breast reduction is nearly always performed as a day surgery procedure, with a same-day admission only required in select cases. Patients are scheduled for an average of 2.7 postoperative visits (range: 2-5). Three surgeons routinely (28.6%) or sometimes (14.3%) offer patients’ nursing care services for drain and/or dressing management. Among those who administer drains, they are removed in the clinic by the surgeons themselves (n = 2) or by community care (n = 3). Four of the 7 surgeons (57.1%) use antibiotics, specifically cefazolin, postoperatively. Only one surgeon (14.3%) provides a prescription for compression stockings, and no surgeons provide a prescription for postoperative anticoagulation at home.

### Cost Variability

#### Healthcare Costs

Healthcare cost variability is summarized in Table 3. The lowest and highest costing providers cost an average of $5156.40 and $7146.41 per patient, respectively, with a cost difference of $2033.95 between the maximalist and minimalist spending surgeons. This variability in cost can be attributed to differences in the amount of OR time booked, the number of preoperative and postoperative visits, and community nursing care usage. A 2-h OR booking (one surgeon) costs $1974 less than a 3-h booking (4 surgeons) due to time-based operating room fees and anesthesiologist billing. While 2 surgeons routinely schedule 2 to 3 perioperative visits, another routinely schedules 4, leading to a cost difference of up to $93 to the healthcare system. Additionally, nursing care usage for postoperative drain/dressing management was associated with an additional spending of $59 for 3 surgeons.

**Table 3.** Healthcare and Patient Cost Variability by Surgeon.

	PS1	PS2	PS3	PS4	PS5	PS6	PS7
Healthcare costs ($)							
*Preoperative costs*							
Clinic Assessments by Surgeon^a^	153.35	106.85 (91.35-122.35)	122.35	122.35	106.85 (91.35-122.35)	106.85 (91.35-122.35)	91.35
Consultation by Anesthesiologist	109.70	109.70	54.85 (0.00-109.70)	109.70	54.85 (0.00-109.70)	54.85 (0.00-109.70)	109.70
*Intraoperative costs* ^b^							
OR cost	4471.58	3577.26	5365.89	5365.89	4471.58	5365.89	5365.89
Surgeon billing costs	944.30	944.30	944.30	944.30	944.30	944.30	944.30
Anesthesiologist billing costs	418.23	325.29	511.17	511.17	418.23	511.17	511.17
*Postoperative costs*							
Clinic follow-ups by surgeon	93.00	93.00	62.00	62.00	62.00	62.00	124.00
Community nursing clinic visits	0.00	0.00	59.44	59.44	29.72 (0.00-59.44)	0.00	0.00
Patient-borne costs ($)							
*Preoperative costs*							
Clinic visits with surgeon^c^; mean (range)	69.00 (21.00-117.00)	34.50 (7.00-78.00)	46.00 (14.00-78.00)	46.00 (14.00-78.00)	34.50 (7.00-78.00)	34.50 (7.00-78.00)	23.00 (7.00-39.00)
Visit with anesthesiologist^c^; mean (range)	23.00 (7.00-39.00)	23.00 (7.00-39.00)	11.50 (0.00-39.00)	23.00 (7.00-39.00)	11.50 (0.00-39.00)	11.50 (0.00-39.00)	23.00 (7.00-39.00)
*Postoperative costs*							
Community home care clinic visits	0.00	0.00	21.50 (7.00-36.00)	21.5 (7.00-36.00)	10.00 (0.00-36.00)	0.00	0.00
Postoperative antibiotics	18.42	0.00	18.42	18.42	0.00	0.00	18.42
Follow-up visits with surgeon^c^	69.00 (21.00-117.00)	69.00 (21.00-117.00)	46.00 (14.00-78.00)	46.00 (14.00-78.00)	46.00 (14.00-78.00)	46.00 (14.00-78.00)	92.00 (28.00-156.00)
Total healthcare costs per patient ($)	**6190.16**	**5156.40 (5140.90-5171.90)**	**7120.00 (7065.15-7174.85)**	**7174.85**	**6087.53 (5987.46-6187.60)**	**7045.06 (6974.71-7115.41)**	**7146.41**
Total costs incurred by patient ($)	**179.42 (67.42-291.42)**	**126.50 (35.00-234.00)**	**143.42 (53.42-249.42)**	**154.92 (60.42-249.42)**	**102.00 (21.00-231.00)**	**92.00 (21.00-195.00)**	**156.42 (60.42-252.42)**

Abbreviations: BBR, bilateral breast reduction; OHIP, Ontario Health Insurance Plan; OR, operating room; PS, plastic surgeon. Bolded values represent total calculated costs.

^a^ Initial consultation visit and any subsequent preoperative follow-ups.

^b^ Calculated based on average OR time booked by surgeon.

^c^ All clinical visits with a physician were assumed to occur in a hospital setting. Costs presented reflect patient transportation costs (driving and parking, or public transit).

*Note:* Costs billed by surgeons for performing the procedure itself are consistent across all surgeons as all surgeons use the same OHIP billing code for BBR, regardless of the OR time booked.

Costs excluded from analysis include those associated with preoperative mammography and liposuction.

#### Patient-Borne Costs

Variability in costs absorbed by patients is summarized in Table 3. Direct patient costs to attend in-person appointments varied according to the number of preoperative and postoperative visits scheduled by their surgeon and/or anesthesiologist. It costs patients an average of $223.95 (range: $117-$291.42) and $49.03 (range: $21-$67.42) to attend hospital appointments and/or community nursing care appointments, respectively. Differences in methods of transportation (ie, driving or public transportation) contribute to variation in patient costs via vehicle wear and tear (ie, $13.60 for an average round-trip distance of 20 km),^8^ parking fees (ie, $23.00 for a 2-h hospital appointment^9^ or $2.00 for a 1-h community nursing care appointment),^10^ or public transportation fares ($6.50 for an adult round-trip fare).^11^ There is a cost difference of up to $270.42 per patient between the highest- and lowest-costing surgeons, reflecting intersurgeon variability.

## Discussion

To our knowledge, this is the first cost analysis study assessing BBR practices among Canadian surgeons practicing in a tertiary care center in Ontario. Variability was identified across multiple areas of practice, with notable differences in the number of pre- and postoperative assessments by a surgeon, completion of a preoperative assessment by an anesthesiologist, length of OR time booked, drain use, and use of community nursing care services for drain care. A significant difference in healthcare spending of over $2000 per patient was identified between plastic surgeons. Similarly, patient-borne costs varied by over $87 per patient. The significant variability in costs—both to the MOH and to patients—can be explained by the aforementioned variations in practice among surgeons.

With a procedure as common as BBR, even minor differences in practice approaches—and the associated cost variability—carry immense implications on healthcare cost at the MOH level. Hypothetically, if we were to assume an average of 5000 BBRs performed annually in Ontario, Canada, an intersurgeon variability of $2034 or higher per patient might contribute to over 10 million dollars in excess spending by the provincial healthcare system.^1^ This observed cost variability highlights the need for standardization of BBR practices.

In this survey, certain practice variations may be explained by differences in surgeons’ preferences, comfort, or experience levels. Differences in OR booking time may be explained by variations in years in practice and area of subspecialty focus, with younger surgeons with a fellowship in a nonbreast focused specialty tending to book longer operating times than a more experienced, breast-focused surgeon. Booking time may also be influenced by patients’ preoperative breast size and a surgeon's willingness to operate on larger-breasted patients, as these cases inherently require longer operative times. A surgeon's technical approach to BBR, including the reduction pattern and pedicle selected, will further influence BBR case time, with some approaches requiring less operative time than others (eg, superior-medial vs wise pattern inferior pedicle) based on the procedural steps involved and depending on patient suitability.^12^ Some variation in operative case length between surgeons is inevitable; however, these differences may be reduced if surgeons adopt technical approaches that maximize efficiency whenever appropriate.

Furthermore, variation may be explained by guidelines that are unclear and/or limited in robustness. For example, the decision to place drains intraoperatively was made by 4 of 7 surgeons. In these cases, drain care and removal was performed either by the surgeon themself or by community nursing care services, thus contributing to additional healthcare costs and patient spending to attend these appointments. Unfortunately, guidelines for drain use in BBR remain unclear.^13^ Although the American Society of Plastic Surgeons (ASPS) guidelines suggest that drain use is not beneficial, a 2024 review of the trials on which these recommendations were based revealed a lack of robustness, thus bringing the strength of this recommendation into question.^13^ In the absence of methodologically robust, evidence-based BBR guidelines, it is difficult to discern whether the added costs associated with drain care are necessary or not. Similarly, guidelines do not provide specific recommendations on the number of perioperative visits with a surgeon or anesthesiologist, further contributing to variability and potential resource waste. Institutional practices may also contribute to variability, as the 2 Hamilton institutions performing BBR differ in their preoperative anesthesia protocols—one requires consultations for all patients, while the other reserves them for higher-risk cases. Although variations in practice are inevitable to some extent, they should be minimized as best as possible to improve patient outcomes and reduce unnecessary costs.^14^

While this study highlights significant cost variability, it is important to note that the true cost differences are likely higher. For example, in a community setting without resident physicians, a surgeon requires a surgical assistant who bills additional fees to the healthcare system—typically adding $225 to $350 per case, depending on the duration of operating room time booked. These additional assistant fees in community settings may, however, be counterbalanced by the costs inherently borne in academic centers, including resident salaries, teaching stipends, and other educational resources. Additionally, according to one surgeon, prior to the COVID-19 pandemic, drain care was provided in the home as opposed to a community clinic, which is associated with even higher healthcare spending. Other costs omitted from the analysis include overhead costs of nursing or home care agencies, administrative costs for patient appointment and OR scheduling, and overhead costs for surgeons practicing in their own office space as opposed to a hospital-based practice. These were selectively omitted, given that the costs specific to BBR patients are difficult to isolate. Anesthesiologist billing costs are further underreported as they were calculated based on the OR time booked, while true billing costs are calculated based on time of patient interaction (including preoperatively and in the postanesthesia care unit). Surgical bra costs borne by patients were additionally omitted, given the significant cost variations between different manufacturers. Finally, 4 surgeons reported offering liposuction as an adjunct to traditional BBR, with one surgeon performing liposuction routinely in all BBR cases. Liposuction-associated costs were excluded from the analysis, given the lack of standardization of billing among cosmetic, non-OHIP-funded procedures. With the addition of liposuction comes an additional thousands of dollars in costs to patients, and the cost-effectiveness of this approach has not yet been described in the literature.

The substantial cost variations in BBR practices highlight the need to identify strategies for reducing these discrepancies moving forward. Unfortunately, the preexisting ASPS Clinical Practice Guidelines on BBR come with their own limitations.^15^ Specifically, they were not designed for use within a single-payer, government-funded healthcare system, and few of their recommendations account for cost—likely due to the limited number of published economic evaluations on BBR. As such, Canadian surgeons face additional challenges in integrating these recommendations into their practice. As well, they do not provide clear recommendations on the areas with the greatest practice variability, such as the number of pre- and postoperative visits with a physician. To minimize variability in practices and subsequently reduce unnecessary spending, there may be a role for the development of a cost-focused Enhanced Recovery After Surgery (ERAS) protocol, with adherence to the ERAS^®^ Society recommendations.^16,17^

At a provincial level, resource waste may be further minimized through evidence-based selection criteria for patients who would qualify for public funding of BBR. In Ontario's single-payer health system, candidacy for OHIP-funded BBR is determined through a MoH approval process that relies largely on patient-reported symptoms documented in the surgeon's application for coverage.^7^ The lack of standardized, measurable selection criteria creates subjectivity when determining which patients are eligible to receive provincial funding. Incorporating a set of evidence-based approval criteria could enhance transparency, optimize resource allocation, and potentially reduce unwarranted approvals.

There were several limitations in the methodology of this cost analysis. First, a series of assumptions was made for the purpose of the analysis. It was assumed that all cases were uncomplicated and did not require hospital admission or additional nursing care visits for wound care complications. The typical number of postoperative nursing care visits was estimated to be 2 (ie, a visit on postoperative days 2 and 4, with drains removed on the second visit). However, in reality, drain removal times are entirely patient-dependent and difficult to accurately predict. An additional limitation was the limited sample size (n = 7) of plastic surgeons who participated in the survey, given the small sample of plastic surgeons practicing within the geographic area (Hamilton, Ontario) who perform BBR. Despite this limited sample, those who participated in the survey represent a diversity of genders, areas of fellowship training, years in practice, and annual BBR caseload, thus increasing the generalizability of the study results. Furthermore, while the length of OR time per case was shown to contribute substantially to cost variations, it is important to note that a surgeon who dedicates more time per case is likely to complete a lower volume of cases per day, thereby incurring lower total costs to the MoH on a given operative day. However, by calculating each surgeon's cost on a per-case basis—rather than a time-based measure such as daily or weekly costs—we aimed to standardize comparisons across surgeons and isolate procedure-specific cost differences. Finally, some costs (eg, community nursing care services) were difficult to determine accurately and adjust for inflation. However, due to the retrospective design of the study and its relatively short 5-year time frame, assessing cost changes over time was not feasible.

Full economic evaluations within BBR are recommended to allow plastic surgeons to make clinical decisions that are the most cost-effective for our patients and healthcare system, taking into account both costs and clinical outcomes.^18,19^ Namely, cost-effectiveness or cost-utility analyses—as opposed to a simple cost-comparison study—would allow us to better decide where to dedicate our limited resources. Although previous economic evaluations in BBR have been published comparing BBR against conservative management,^20,21^ as well as specific surgical approaches (vertical scar vs inverted T-shape reduction),^22^ further evaluations are necessary to address the prevailing variability in practices.

## Conclusion

Bilateral breast reduction is a widely chosen procedure to enhance the quality of life in patients with breast hypertrophy, though practice patterns vary. Within Hamilton, Ontario, significant variability is noted between surgeons’ preferences, specifically with regard to the number of pre- and postoperative visits, drain use (and the associated community nursing care services), OR time booked, and the involvement of a preoperative assessment by anesthesia colleagues. This study highlights the significant healthcare spending discrepancies of over $2000 across different surgeons’ practices—a result of variations in practice. Variable practices also contribute to differences in patient spending. While variability in surgical practice is not problematic in isolation, it must be addressed when precious resources are being wasted, especially considering our current economic climate. Thus, efforts should be made to (1) characterize the cost-effectiveness of variable BBR practices through full economic analyses, and (2) standardize BBR practices in a manner that considers costs alongside patient outcomes.

## Supplemental Material

sj-docx-1-psg-10.1177_22925503251386770 - Supplemental material for The Impact of Breast Reduction Practice Variation in Hamilton, Ontario: A Cost Analysis Supplemental material, sj-docx-1-psg-10.1177_22925503251386770 for The Impact of Breast Reduction Practice Variation in Hamilton, Ontario: A Cost Analysis by Tara Behroozian, MD, Alice Wang, MD(C), Lucas Gallo, MD, MSc, PhD(C), Justin Haas, MD, GDip, Helene Retrouvey, MDCM, PhD, FRCS(C), Ammara Ghumman, MD, MSc, FRCS(C), and Achilles Thoma, MD, MSc, FRCS(C) FACS in Plastic Surgery
