# Supplementary material for: The Impact of Breast Reduction Practice Variation in Hamilton, Ontario: A Cost Analysis
Source: Plast Surg (Oakv). 2025 Oct 21:22925503251386770. Online ahead of print. doi: 10.1177/22925503251386770 (PMC12540353; doi:10.1177/22925503251386770)
Supplement: sj-docx-1-psg-10.1177_22925503251386770 - Supplemental material for The Impact of Breast Reduction Practice Variation in Hamilton, Ontario: A Cost Analysis [file sj-docx-1-psg-10.1177_22925503251386770.docx]

**APPENDIX A: SURVEY**

***Practice Profile***

1. Please indicate your gender identity:
   1. Male
   2. Female
   3. Other
   4. Prefer not to disclose
2. How many years have you been in practice?
3. If you completed fellowship training, what area are you fellowship-trained in? (optional)
4. On average, how many BBRs do you perform annually? (optional)
5. Do you perform OHIP-funded or private BBRs?
   1. OHIP only
   2. Private only
   3. Both

***Preoperative Care***

1. Do you operate on smokers?
   1. Yes
   2. No
   3. Sometimes (specify under what circumstances)
2. Do you instruct patients to stop smoking before BBR?
   1. Yes
      1. If yes, at what time point before surgery?
         1. 2 weeks before BBR
         2. 3 weeks before BBR
         3. 4 weeks before BBR
         4. Other
   2. No
3. Do you recommend patients to lose weight preoperatively?
   1. Yes (specify weight loss goals; any BMI threshold?)
      1. If yes, do you re-assess patients after weight loss for weight stabilization prior to surgery?
   2. No
4. How many times is the patient seen before the day of operation? _____
5. Is a pre-operative assessment completed by the anesthesia team?
   1. Yes
   2. No
   3. Sometimes (specify under what circumstances)
6. Do you conduct a general physical examination (e.g. cardiac, respiratory, and abdominal) in addition to the breast examination prior to surgery?
   1. Yes
   2. No
   3. Sometimes (specify under what circumstances)

1. Do patients undergo pre-operative mammography?
2. Yes

**○** If so, which patients (may select multiple)

■ All patients

■ Age 40+

■ Age 45+

■ Age 50+

■ Family history of breast cancer

■ Palpable lump on physical exam

■ Most recent mammogram over 2 years ago

1. No
2. If they undergo pre-operative mammography, what is the age threshold?
3. Older than 40 years
4. Older than 50 years.
5. Do you offer liposuction to patients?
   1. Yes, all patients
   2. Yes, some patients
      1. If so, under what circumstances?
   3. No

***Intraoperative Care***

1. Do you use drains?
   1. Yes
   2. No
   3. Sometimes (specify under what circumstances)

1. Do you use tumescent fluid in the breast?
   1. Yes
   2. No
   3. Sometimes (specify under what circumstances)

1. Do you use antibiotics intraoperatively/pre-operatively?
   1. Yes (specify which)
   2. No

1. Do you use venous thromboembolism prophylaxis?
2. Yes
   1. If yes, using which method:
      1. Ted stockings
      2. Other compression devices
      3. Subcutaneous heparin
      4. Combination of above
3. No

1. How long do you normally book BBR in the OR schedule? _________

1. What percentage of your breast reductions are:
2. Liposuction-assisted
   1. If so, using liposuction for (select all that apply):
      1. Breast reduction itself
      2. Lateral chest wall
      3. Other (specify)
3. Liposuction-only

***Postoperative Care***

1. What percentage of your patients are done as:
2. Day surgery
3. Same day admission
4. List your routine post-operative follow-up protocol: _________

1. Are patients given home care services routinely?
2. Yes
3. No
4. Sometimes (specify under what circumstances)
5. If you use drains routinely, who performs the drain removal and when? __________

1. Do you use antibiotics post-operatively?
   1. Yes (specify which)
   2. No
2. Are patients given a prescription for compression stockings?
   1. Yes
   2. No
3. Are patients given a prescription for post-operative anticoagulation at home?
4. Yes
5. No

***Other***

1. Do you feel you are adequately remunerated for this procedure (excluding the addition of liposuction)?
   1. Yes
   2. No

1. Do you feel the current OHIP fee schedule provides reasonable compensation?
2. Yes
3. No
4. Do you routinely book BBR as a combined OHIP and delisted procedure?
5. Always
6. Often
7. Rarely
8. Never

**APPENDIX B: HEALTHCARE AND PATIENT-BORNE COSTS**

| **Preoperative healthcare costs** | **Unit cost ($)** | **Source** |
| --- | --- | --- |
| *Surgeon* | *91.00* | Ontario Ministry of Health “Schedule of Benefits For Physician Services Under the Health Insurance Act”[^1^](https://www.zotero.org/google-docs/?tjRviQ) |
| Consultation | 91.00 |  |
| *Anesthesia* | *109.70* |  |
| Consultation | 109.70 |  |
| **Intraoperative healthcare costs** | **Unit cost ($)** | **Source** |
| *Operating room* | *1,788.63* | Ontario Case Costing Initiative, 2023-24 (through Finance Department of tertiary academic care centre) |
| Labour | 609.80 |  |
| Supplies | 1,047.28 |  |
| Other | 131.55 |  |
| *Anesthesia* | *Variable total*^†^ |  |
| Unit fee | 15.49 | Ontario Ministry of Health “Schedule of Benefits For Physician Services Under the Health Insurance Act”[^1^](https://www.zotero.org/google-docs/?HTzpzm) |
| *Surgeon* | *944.30* |  |
| Remuneration for procedure | 944.30 |  |
| **Postoperative healthcare costs** | **Unit cost ($)** | **Source** |
| *Surgeon* | *31.00* |  |
| Post-operative assessment | 31.00 | Ontario Ministry of Health “Schedule of Benefits For Physician Services Under the Health Insurance Act”[^1^](https://www.zotero.org/google-docs/?nJq76A) |
| *Keflex prescription* | *18.42* |  |
| Formulary cost | 4.85 | Ontario Drug Benefit Formulary[^2^](https://www.zotero.org/google-docs/?6wn7su) |
| Local pharmacy dispensing fee | 13.57 | Rexall Pharmacy Group ULC |
| *Community nursing clinic visits* | *59.44* |  |
| Nursing care | 59.44 (per hour) | Study on wound care management in Niagara, Ontario (2008)[^3^](https://www.zotero.org/google-docs/?jEZ9Tz) |
| **Patient-borne costs** | **Unit cost ($)** | **Source** |
| *Transportation to one appointment via public transit* | *6.50* |  |
| Public transit fare (round trip) | 6.50 | Hamilton Street Railway (HSR)[^4^](https://www.zotero.org/google-docs/?2yY8kh) |
| *Transportation to one appointment via personal vehicle* | *18.00 - 39.00* |  |
| Hospital parking* | 23.00 | Parking Services office of hospital[^5^](https://www.zotero.org/google-docs/?m16M1Y) |
| Community nursing care clinic parking** | 2.00 | City of Hamilton[^6^](https://www.zotero.org/google-docs/?mpQ3zB) |
| Fuel*** | 2.40 | CBC Gas Pricing Widget for Hamilton, ON[^7^](https://www.zotero.org/google-docs/?9sPV8z) |
| Vehicle wear and tear**** | 13.60 | Canadian Revenue Agency Automobile Allowance Rate[^8^](https://www.zotero.org/google-docs/?785U7v) |
| ^†^ Total number of units billed by anesthesiologist varies based on amount of time spent with patient  *Assuming a 2-hour appointment duration (including time spent waiting and with a provider), with a daily rate of $23.00  ** Assuming a 1-hour appointment duration (including time spent waiting and with a provider)  ***Assuming an average round-trip distance of 20 km and an average fuel efficiency of 8 L/100 km,[^9^](https://www.zotero.org/google-docs/?wlnKIe) with gas priced at $1.50 per litre,[^7^](https://www.zotero.org/google-docs/?0lA8Xs) the fuel cost would be approximately $2.40.​  **** Using the Canada Revenue Agency's 2024 automobile allowance rate of $0.68 per km, the cost for an average round-trip distance of 20 km would be $13.60.​ | | |

**References**

[1.](https://www.zotero.org/google-docs/?9lsBkN) [Ministry of Health. Schedule of Benefits: Physician Services Under the Health Insurance Act. Published online February 2025.](https://www.zotero.org/google-docs/?9lsBkN)

[2.](https://www.zotero.org/google-docs/?9lsBkN) [Ontario Drug Benefit Formulary. Ontario Drug Benefit Formulary/Comparative Drug Index. Accessed July 14, 2025. https://www.formulary.health.gov.on.ca/formulary/results.xhtml?q=%22Cephalexin%22&type=2](https://www.zotero.org/google-docs/?9lsBkN)

[3.](https://www.zotero.org/google-docs/?9lsBkN) [Hurd T, Zuiliani N, Posnett J. Evaluation of the impact of restructuring wound management practices in a community care provider in Niagara, Canada. *Int Wound J*. 2008;5(2):296-304. doi:10.1111/j.1742-481X.2008.00484.x](https://www.zotero.org/google-docs/?9lsBkN)

[4.](https://www.zotero.org/google-docs/?9lsBkN) [City of Hamilton. Fares & Photo IDs. City of Hamilton. Accessed July 14, 2025. https://www.hamilton.ca/home-neighbourhood/hsr/fares/fares-photo-ids](https://www.zotero.org/google-docs/?9lsBkN)

[5.](https://www.zotero.org/google-docs/?9lsBkN) [St. Joseph’s Healthcare Hamilton. Parking at St. Joseph’s Healthcare Hamilton. St. Joseph’s Healthcare Hamilton. Accessed July 14, 2025. https://www.stjoes.ca/patients-visitors/your-visit-or-stay/parking?resourceID=404&lang=EN](https://www.zotero.org/google-docs/?9lsBkN)

[6.](https://www.zotero.org/google-docs/?9lsBkN) [City of Hamilton. Parking Meters & Pay Machines. City of Hamilton. Accessed July 14, 2025. https://www.hamilton.ca/home-neighbourhood/getting-around/parking/parking-meters-pay-machines](https://www.zotero.org/google-docs/?9lsBkN)

[7.](https://www.zotero.org/google-docs/?9lsBkN) [CBC. Gas Pricing Widget - Hamilton. Accessed July 14, 2025. https://www.cbc.ca/news2/interactives/gas-pricing-widget/index.html?type=lowest&id=23](https://www.zotero.org/google-docs/?9lsBkN)

[8.](https://www.zotero.org/google-docs/?9lsBkN) [Canada Revenue Agency. Motor vehicle provided by the employer. Canada Revenue Agency. September 21, 2005. Accessed July 14, 2025. https://www.canada.ca/en/revenue-agency/services/tax/businesses/topics/payroll/benefits-allowances/automobile/automobile-motor-vehicle-benefits.html](https://www.zotero.org/google-docs/?9lsBkN)

[9.](https://www.zotero.org/google-docs/?9lsBkN) [Natural Resources Canada. Fuel consumption ratings search tool. Natural Resources Canada. Accessed July 14, 2025. https://fcr-ccc.nrcan-rncan.gc.ca/en](https://www.zotero.org/google-docs/?9lsBkN)
